# Overcoming the trade‐off between grain weight and number in wheat by the ectopic expression of expansin in developing seeds leads to increased yield potential

**DOI:** 10.1111/nph.17048

**Published:** 2020-12-04

**Authors:** Daniel F. Calderini, Francisca M. Castillo, Anita Arenas‐M, Gemma Molero, Matthew P. Reynolds, Melanie Craze, Sarah Bowden, Matthew J. Milner, Emma J. Wallington, Adam Dowle, Leonardo D. Gomez, Simon J. McQueen‐Mason

**Affiliations:** ^1^ Institute of Plant Production and Protection Universidad Austral de Chile Campus Isla Teja Valdivia 5090000 Chile; ^2^ Institute of Biochemistry and Microbiology Faculty of Sciences Universidad Austral de Chile Valdivia 5090000 Chile; ^3^ International Maize and Wheat Improvement Center (CIMMYT) El Batán Texcoco CP 56237 Mexico; ^4^ NIAB 93 Lawrence Weaver Road Cambridge CB3 0LE UK; ^5^ CNAP Biology Department University of York Wentworth Way, Heslington York YO10 5YW UK

**Keywords:** expansin protein, grain number, grain weight, grasses, pericarp, trade‐off, transgenic

## Abstract

- Wheat is the most widely grown crop globally, providing 20% of all human calories and protein. Achieving step changes in genetic yield potential is crucial to ensure food security, but efforts are thwarted by an apparent trade‐off between grain size and number. Expansins are proteins that play important roles in plant growth by enhancing stress relaxation in the cell wall, which constrains cell expansion.
- Here, we describe how targeted overexpression of an α‐expansin in early developing wheat seeds leads to a significant increase in grain size without a negative effect on grain number, resulting in a yield boost under field conditions.
- The best‐performing transgenic line yielded 12.3% higher average grain weight than the control, and this translated to an increase in grain yield of 11.3% in field experiments using an agronomically appropriate plant density.
- This targeted transgenic approach provides an opportunity to overcome a common bottleneck to yield improvement across many crops.

## Abstract 2

See also the Commentary on this article by Cosgrove, **230**: 403‐405.

## Introduction

Increasing wheat yield is a global priority for food security (Foulkes *et al*., 2011), since this crop provides *c*. 20% of calories and protein in human diets. However, rates of genetic gains in grain yield (GY) potential have decreased since the Green Revolution, and further GY improvement requires new approaches (Foulkes *et al*., 2011; Molero *et al*., 2019). In the past, GY has been consistently increased by higher grain number (GN) per unit area; however, the trade‐off between average grain weight (GW) and GN has become a bottleneck for improving GY, as demonstrated by recent studies in a wide range of wheat genotypes that included elite materials from the International Maize and Wheat Improvement Center (CIMMYT) (Quintero *et al*., 2018; Molero *et al*., 2019; Rivera‐Amado *et al*., 2019).

Increasing individual GW has the potential to improve wheat yield; however, attempts to increase GY by increasing grain size have been hampered by the negative association between GW and GN (Bustos *et al*., 2013; Quintero *et al*., 2018; Molero *et al*., 2019). For example, recurrent selection for higher GW in wheat breeding programmes showed that increases of up to 32% in this trait were completely offset by reductions in GN (Wiersma *et al*., 2001). More recently, genetic studies have been used to identify quantitative trait loci (QTLs) associated with grain size in wheat and other crops (e.g. Gross *et al*., 2003; Simmonds *et al*., 2014; Griffiths *et al*., 2015). For example, Brinton *et al*. (2017) showed a 6.9% increase in GW in near isogenic lines of wheat carrying a QTL on chromosome 5A affecting grain size; and Wang *et al*. (2018) reached the highest increase of GW in triple mutant lines of the *TaGW2* gene (*c*. 20%). However, in these and other cases, increases in GW had little impact on GY due to the trade‐off between GW and GN (Song *et al*., 2007; Zhang *et al*., 2018). Remarkably, this trade‐off is not due to restricted photoassimilation restriction after anthesis (Slafer & Savin, 1994; Borrás *et al*., 2004; Reynolds *et al*., 2009; Quintero *et al*., 2018). Indeed, in an experiment using doubled haploid wheat lines from a cross designed to complement high GN with large seed size, radiation use efficiency increased in response to sink strength during grain filling in comparison with the parental cultivars (Bustos *et al*., 2013). This indicates that breaking the negative association between GN and GY does not require increased photosynthesis during grain growth to increase GY. As source limitation cannot account for the apparent trade‐off between GW and GN, other limiting factors must be at play.

**Fig. 1:**
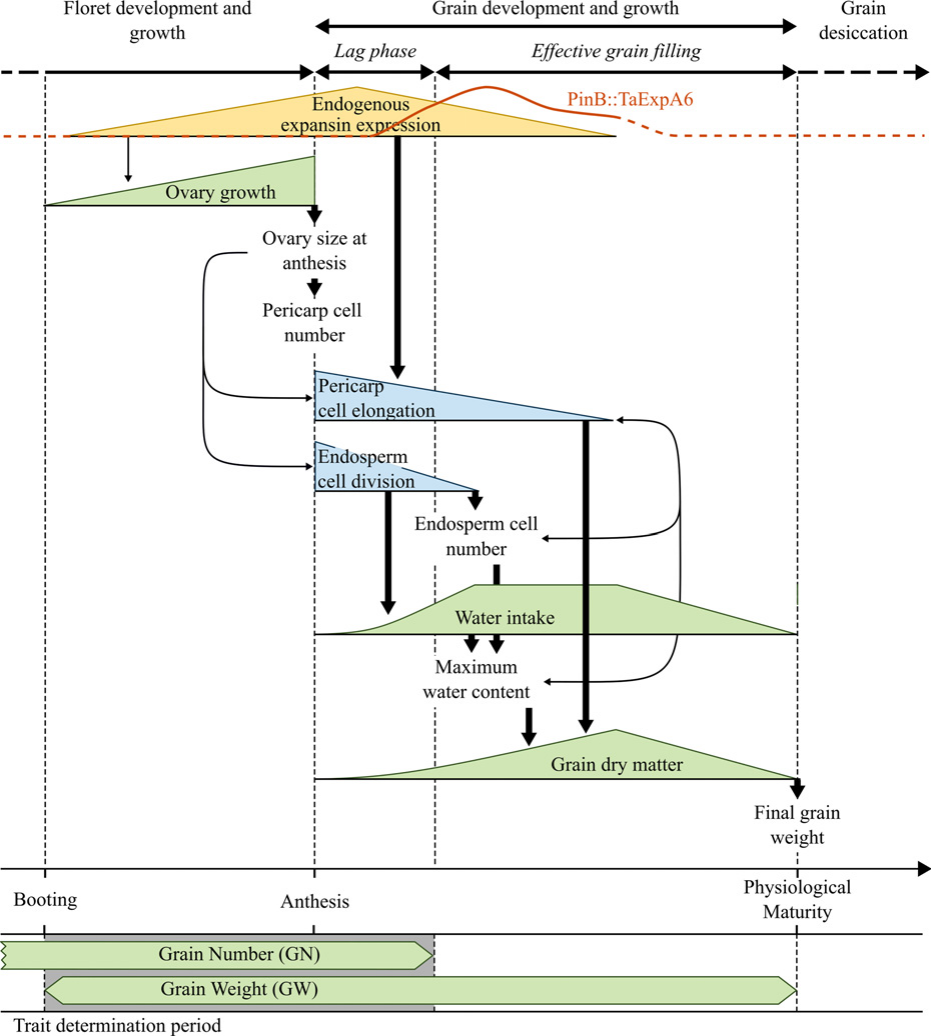
Schematic description of processes and traits of grain weight determination in wheat from booting to maturity. The gene expression of the recombinant *PinB::TaExpA6* and its apparent dynamic is shown. At the bottom of the scheme, the overlap between grain number and grain weight determination from booting to the end of the lag phase is shown. Wide arrows show the main links between processes/traits, and narrow arrows indicate indirect links between processes/traits.

The potential weight of grains is determined early in development, in a period starting just before anthesis and continuing until just beyond grain setting (Calderini *et al*., 1999, 2001; Ugarte *et al*., 2007; Brinton & Uauy, 2019). Grain expansion and grain filling are separated in time, with the ovary expansion preceding grain filling (Calderini *et al*., 1999; Brinton & Uauy, 2019). Grain growth (i.e. grain enlargement and dry matter accumulation) involves the coordinated expansion of the maternally derived pericarp and the seed endosperm, and is almost complete at the time when storage deposition begins (Fig. 1). The expansion of these tissues determines the holding capacity of the seed for storage reserves, and therefore the potential final GW (Brinton & Uauy, 2019; Herrera & Calderini, 2020). Therefore, it seems possible that genetic manipulation of grain expansion in a spatiotemporal manner could increase the rate or duration of this growth phase in grain development and lead to increased yield potential.

In plants, cells grow through turgor‐driven expansion that is constrained by the cell wall (Cosgrove, 2018). Cell‐wall extensibility is under dynamic control in plant cells, and expansins play a key role by inducing the relaxation of the stress that is generated in the cell wall through the action of turgor pressure (McQueen‐Mason *et al*., 1992). Expansin manipulation can lead to changes in growth and development (Fleming *et al*., 1997; Pien *et al*., 2001; Rochange *et al*., 2001; Choi *et al*., 2003). These cell‐wall proteins appear to act by disrupting noncovalent associations between cellulose and matrix polysaccharides in the plant cell wall, allowing the polymers to slip relative to one another, relaxing stress in the wall and allowing it to extend (McQueen‐Mason & Cosgrove, 1994). Expansins fall into two well‐separated groups, designated as α and β‐expansins, based on sequence homology and activity; with α‐expansins more clearly associated with cell expansion and growth (Cosgrove, 2018).

We previously revealed an association between grain expansion and the expression of α‐expansins in wheat, and suggested that the expansin manipulation might provide a way to increase GW (Lizana *et al*., 2010). In the work presented here, we increased the amount of α‐expansin in developing grains, by the ectopic expression of *TaExpA6* (an expansin gene normally expressed in wheat roots) under control of a grain‐specific gene promoter, and show that this can lead to increased GW without a negative impact on GN and, in turn, to improve GY.

## Materials and Methods

### Genetic constructs assembly

For this work, we transformed wheat with a construct containing the *TaExpA6* coding sequence (CDS) under the control of wheat *puroindoline‐b* (*PinB*) gene promoter, which drives transcription restricted to the endosperm, aleurone, and pericarp layers in developing seeds, but not in the embryo (Gautier *et al*., 1994; Digeon *et al*., 1999). We verified that the expression of the *TaPinB* gene is specific to the tissues mentioned using the wheat expression browser (Ramírez‐González, *et al*., 2018). The intermediate cassette containing the *TaPinb‐*promoter (refseq v.1.1: *TraesCS7B02G431200*) controlling the *TaExpA6* CDS was recombined into the binary vector pRLF12‐R1R2 to create pEW279‐Exp, using a Gateway LR Clonase II kit (Thermo Fisher Scientific, Loughborough, Leicestershire, UK). Supporting Information Fig. S1 shows the transfer DNA (T‐DNA) region of this construct. The plasmid was electro‐transformed into *Agrobacterium tumefaciens* strain EHA105 (Hood *et al*., 1993). The sequence of *TaExpA6* gene was obtained from GenBank accession no. *AY543532* (www.ncbi.nlm.nih.gov/nuccore/AY543532) and verified in the recently released genome sequence of Chinese Spring variety, refseq v.1.1 (*TraesCS4A02G034200*) (Appels *et al*., 2018).

### Wheat transformation and subsequent wheat generations

Transformation of immature embryos isolated from spring wheat cv Fielder was carried out by co‐cultivation of pretreated immature wheat embryos with *Agrobacterium* containing pEW279‐Exp at 23°C in the dark for 2 d (Ishida *et al*., 2014). Following removal of the embryonic axis, tissue culture was performed essentially as described previously (Risacher *et al*., 2009). From 37 regenerated wheat plants, DNA was isolated using the DNA extraction protocol described by Howells *et al*. (2018), and the plants were confirmed as transformant by PCR amplification of the transgene using primers Exp6‐forward (5′‐CCG TTC TCG CGT TCT GCT TCGT‐3′) and NosT‐reverse (5′‐CGA TCG GGT GAA ATT CGG ATCC‐3′) using FastStart Taq polymerase (Thermo Fisher Scientific) with an annealing temperature of 53°C. A transformation efficiency of 30.6% was achieved with this construct, calculated as the percentage of wheat embryos from which a transformed wheat plant was regenerated. It is important to point out that the control wheat plants used in the experiments correspond to cv Fielder that underwent the same tissue culture process as the transformed lines.

From each wheat transformant plant (T_0_), 10 individuals plants were grown in pots in a glasshouse (10 individual T_1_ plants from 37 lines, total 370 plants) at The University of York, and the presence of *TaExpA6* was checked by PCR using a forward primer Ta‐*PinB*‐F (5′‐ACAACACACAATGGTAGGCAAA‐3) and reverse primer *TaExpA6*‐R (5′‐ GGTCCCCTTCACCGACAT‐3′). T‐DNA copy number was determined by quantitative PCR (qPCR) assay of the *nptII* gene relative to a single‐copy wheat gene amplicon, normalized to a known single‐copy wheat line in T_0_ and T_1_ plants (Table S1). In the next generations (T_2_, T_3_, T_4_) we carried out genomic DNA extraction from leaves using the CTAB method standardized protocol and copy number determination was performed by IDna Genetics (UK) (https://www.idnagenetics.com/) (Table S2).

The multiplication of T_2_ seed was also carried out in glasshouses in 2015–2016. During the vegetative stage, leaves were sampled for PCR analysis to identify homozygous lines. Lack of negative segregants among the sampled progeny (at least 10 individuals sampled per line) was used as an indicator of homozygosis. Twenty‐four homozygous lines were selected to be evaluated in field experiments (T_3_ and T_4_ generations).

### Experiments and field conditions (T_3_ and T_4_ generations)

Two field experiments were carried out at the Experimental Station of the Universidad Austral de Chile in Valdivia (39°47′S, 73°14′W). Experiment 1 was a low plant density (LPD) experiment with T_3_ lines during the 2016–2017 growing season. The aim of the LPD experiment was to increase the seed bank and evaluate the performance of lines under field conditions. Experiment 2 was sown at regular plant density (RPD) with four selected T_4_ lines in the 2018–2019 season.

In the LPD experiment, 24 wheat transformed lines and the control spring cv Fielder were sown at a plant density of 44 m^−2^. Plots were 1.5 m long and 0.6 m wide, with five rows 0.15 m apart and 0.15 m between seeds in each row. In the RPD experiment, four selected homozygous transformant lines and the control were sown at farmer’s conventional plant density of 300 m^−2^. In these experiments, the same plot dimensions and row spacing were used, but seed was sown at 0.022 m intervals. In both experiments, additional rows were sown rounding each plot to avoid border effects. The experiments were arranged in a complete randomized block design with three replications. All plots were subjected to optimal agronomical management to assure high potential yield conditions. To this end, plots were fertilized, drip irrigation was applied to avoid water stress and weeds, insects and diseases were prevented or controlled in both experiments.

In the RPD experiment, the four transformant lines were selected based on performance in the LPD experiment, low construct copy numbers, and levels of transgene expression recorded in the previous generation (Table S1).

### Crop phenology and measurements

Crop phenology was recorded following the decimal code scale (Zadoks *et al*., 1974) at each plot. At harvest, a 1 m length of the central row was sampled in each plot to determine GY and components in both experiments. Plant samples were oven‐dried at 60°C for 48 h for DW. GN and average GW were measured. GY per plant was calculated in the LPD experiments and GY per square metre in the RPD experiments. Grain dimensions (length, width, and area) were recorded using a Marvin seed analyzer (Marvitech GmbH, Wittenburg, Germany) after grains were oven‐dried at 60°C for 48 h.

### RNA extraction and expression analyses by quantitative reverse transcription quantitative PCR

Grains of grain position one and two (G1, G2) from central spikelets of two main stem spikes (eight grains in total each replicate) were sampled at 5, 15, and 25 d post‐anthesis (DAA) in LPD experiment and at 5, 10, 15, 20 and 25 DAA in the RPD experiment for RNA extraction and gene expression analysis. Grains were sampled and frozen in liquid nitrogen (N_2_). Total RNA was extracted using NucleoSpin™ columns (Macherey‐Nagel, Düren, Germany) following the manufacture’s protocol and standardizing the RNA extraction protocol based on Sangha *et al*. (2010).

The *TaExpA6* expression in grains was assessed by quantitative reverse transcription PCR (RT‐qPCR). Complementary DNA was synthesized from 500 ng RNA (pretreated with DNaseI (Invitrogen) using ImProm‐II™ Reverse Transcription System). The qPCR reactions, with a final volume of 25 μl, were performed using the Brilliant II SYBR Green PCR Master Mix (Stratagene; Agilent Technologies, Santa Clara, CA, USA), 0.2 μM and primers Trangene*TaExpA6*_F1 (5′‐ATCTCCACCACCACCAAAACA‐3′) and Transgene*TaExpA6*_R1 (5′‐GAAGCAGAACGCGAGAACGG‐3′). No‐template and no‐transcriptase controls were included to detect genomic DNA contamination. The transcript abundance of the *TaExpA6* gene in grains was determined using the method of Pfaffl (2001), where the ubiquitin‐conjugating enzyme (*TraesCS4A02G414200*) gene was used as an internal control, using the primers *Forward* (5′‐ CGGGCCCGAAGAGAGTCT‐3′) and *Reverse* (5′‐ ATTAACGAAACCAATCGACGGA‐3′). Fluorescence raw data were analysed with linregpcr software for quantification analysis of gene expression (Ruijter *et al*., 2009).

### Protein extraction and LC–MS proteomics

We extracted total proteins from wheat grains of the position two of the central spikelets at 15 DAA. Grains were ground with liquid N_2_, and 40 mg of pulverized grains were added to 100 μl of sodium dodecyl sulphate (SDS) polyacrylamide gel electrophoresis (PAGE) loading protein buffer 2× at 95°C for 10 min and centrifuged at 13 000 ***g*** for 10 min for protein extraction. Then, 5 µl aliquots were loaded per lane in a 12% SDS‐PAGE. Gels were stained with SimpleBlue SafeStain (safe Coomassie G‐250). In‐gel tryptic digestion was performed, as previously described (De Pablos *et al*., 2019). Peptides were loaded onto an UltiMate 3000 RSLCnano HPLC system (Thermo Fisher Scientific) equipped with a PepMap 100 Å C18, 5 µm trap column (300 µm × 5 mm; Thermo Fisher Scientific) and a PepMap, 2 µm, 100 Å, C18 EasyNano nanocapillary column (75 µm × 150 mm; Thermo Fisher Scientific) and separated as previously described (De Pablos *et al*., 2019), with the exception that the following gradient was used: 3–10% B over 8 min, 10–35% B over 125 min, 35–65% B over 50 min, 65–99% B over 7 min, and then proceeded to wash with 99% solvent B for 4 min. The nanoLC system was interfaced with an Orbitrap Fusion hybrid mass spectrometer (Thermo Fisher Scientific) with an EasyNano ionization source (Thermo Fisher Scientific). Positive electrospray ionization MS and MS^2^ spectra were acquired using xcalibur software (v.4.0; Thermo Fisher Scientific) as previously described (De Pablos *et al*., 2019). Tandem mass spectra were searched against the *Triticum aestivum* subset of the UniProt database appended with the sequence of *TaEXPA6* using the mascot program (v.2.6.1; Matrix Science Ltd, London, UK). Search criteria specified: missed cleavages, 1; fixed modifications, carbamidomethyl (C); variable modifications, oxidation (M); peptide tolerance, 3 ppm; MS/MS tolerance, 0.5 Da. Results were filtered to 2% false discovery rate against a reversed database and required a minimum of two unique peptides per protein. Label‐free peptide quantification was extracted from aligned precursor ion areas using progenesis QI (v.2.2.; Waters, Hertfordshire, UK).

### Statistical analysis of data

ANOVA was applied when multiple groups of data were compared, followed by pairwise comparisons between the control and each T line (Fisher’s least significant difference test *post hoc*) to evaluate significant differences (*P* < 0.10, *P* < 0.05, *P* < 0.01 and *P* < 0.001) using the Statistica 7 software. Regression analyses were used to evaluate the degree of association between variables.

## Results

We generated transgenic wheat plants expressing *TaExpA6*, an α‐expansin normally expressed in wheat roots (according to wheat‐expression browser: Ramírez‐González *et al*., 2018), under the control of the wheat puroindoline‐b (PinB) gene promoter (*TraesCS7B02G431200*), which drives transcription restricted to the endosperm, aleurone, and pericarp layers in developing seeds, but not in the embryo (Gautier *et al*., 1994; Digeon *et al*., 1999). We confirmed that the expression of the PinB gene is negligible outside the tissues mentioned using the wheat expression browser (Ramírez‐González, *et al*., 2018). We further confirmed this by RT‐qPCR, and no expression of the transgene was found in roots and vegetative tissues (data not shown). Homozygous T_2_ lines were selected, and T_3_ and T_4_ generations were evaluated in field experiments at different plant densities. Low‐density plantings (44 m^−2^) were carried out during the 2016–2017 growing season using T_3_ seed, whereas a more typical agronomic planting density (300 m^−2^) experiment was carried out using T_4_ seed in the 2018–2019 growing season in Valdivia (Chile). Transgene expression in developing seeds was assessed by RT‐qPCR, and the levels of recombinant *TaExpA6* protein were assessed by semi‐quantitative proteomic analysis.

### *TaExpA6* expression and protein abundance in growing seeds of T_3_ and T_4_ transgenic lines and segregating wild‐type plants

**Fig. 2:**
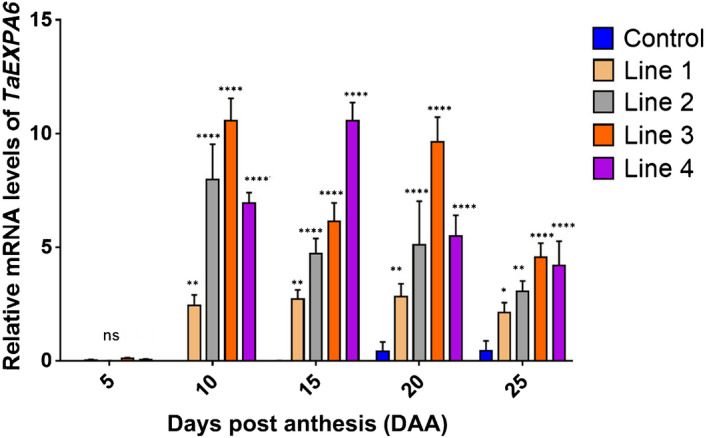
Relative expression of *TaExpA6* in grains at 5, 10, 15, 20 and 25 d after anthesis (DAA) in the field experiment at regular agronomical plant density of 300 m^−2^ assessed by quantitative reverse transcription PCR. Control line corresponds to spring wheat cv Fielder that has undergone the same tissue culture process as the transformed lines. Asterisks indicate significant differences by pairwise comparisons between each line and the control (Fisher’s least significant difference test *post hoc*): *, *P* < 0.10; **, *P* < 0.05; ***, *P* < 0.01; ****, *P* < 0.001; ns, not significant. All data are shown as mean and SE. mRNA, messenger RNA.

**Fig. 3:**
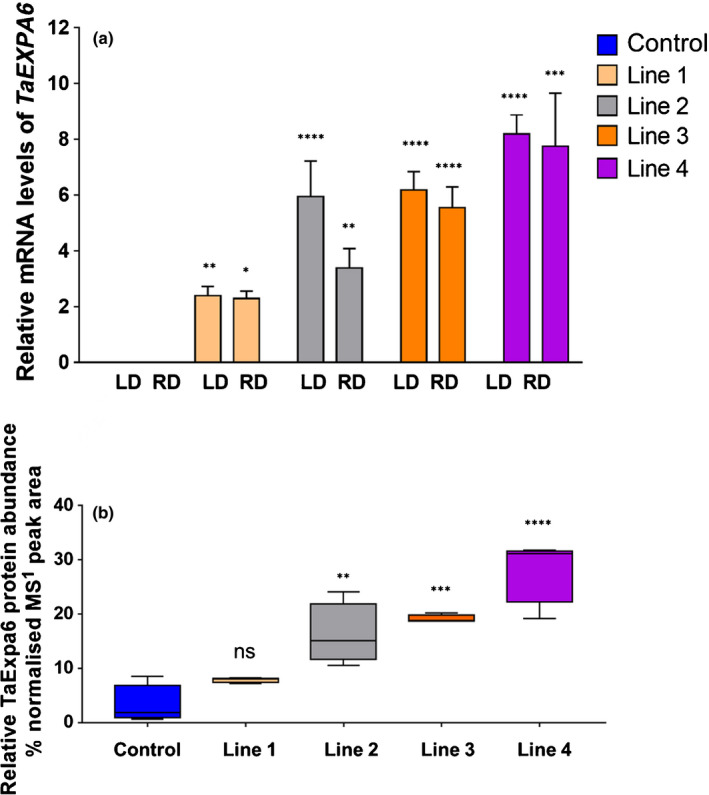
Expression and protein abundance of *TaExpA6* in grains. Control line corresponds to spring wheat cv Fielder that has undergone the same tissue culture process as the transformed lines. (a) Relative messenger RNA (mRNA) levels of the *TaExpA6* transgene assessed by quantitative reverse transcription PCR in grains at 15 d after anthesis (DAA) in the control and transformed lines 1, 2, 3 and 4 from experiment with low density (LD) and regular density (RD) planting. (b) Relative protein abundance as assessed by LC–MS/MS analysis at 15 DAA in the control and transgenic lines 1, 2, 3 and 4 at regular agronomical plant density. Asterisks indicate significant differences by pairwise comparisons between each line and the control (Fisher’s least significant difference test *post hoc*): *, *P* < 0.10; **, *P* < 0.05; ***, *P* < 0.01; ****, *P* < 0.001; ns, not significant. All data are shown as mean and SE.

Twenty‐four transgenic lines were generated and evaluated in a field experiment together with wild‐type of cv Fielder that had been through the same tissue culture cycle as the transformants (control) at LPD (44 m^−2^), aimed also at bulking seed for further studies. Based on the transcript abundance and transgene copy number (Fig. S2; Tables S1, S2), four transformed lines were chosen for further analysis. Transgene transcripts of *TaExpA6* were not detected in growing seeds at 5 DAA and showed a peak in abundance between 10 and 20 DAA (Fig. 2). In both experiments, all the transgenic lines showed *TaExpA6* transcripts, which were not apparent in the control (Fig. 3a). Transgenic lines 4 and 3 showed the highest relative transcript abundance (Fig. 3a).

The presence of *TaExpA6* protein was assessed by extracting total proteins from developing seeds at 15 DAA (see Materials and Methods section). Lizana *et al*. (2010) showed that several α‐expansin genes are normally expressed in developing seeds, and distinguishing between these closely related proteins is difficult using immunological methods. Instead, we used LC–MS/MS proteomic methods, which allowed unambiguous protein identification, as well as the determination of relative protein abundance. The abundance of the recombinant *TaExpA6* protein showed good agreement with the observed transgene expression levels in transgenic lines, with lines 3 and 4 showing the highest recombinant protein abundance (Fig. 3b).

### *TaExpA6* ectopic expression increases grain size and weight in transgenic wheat

**Fig. 4:**
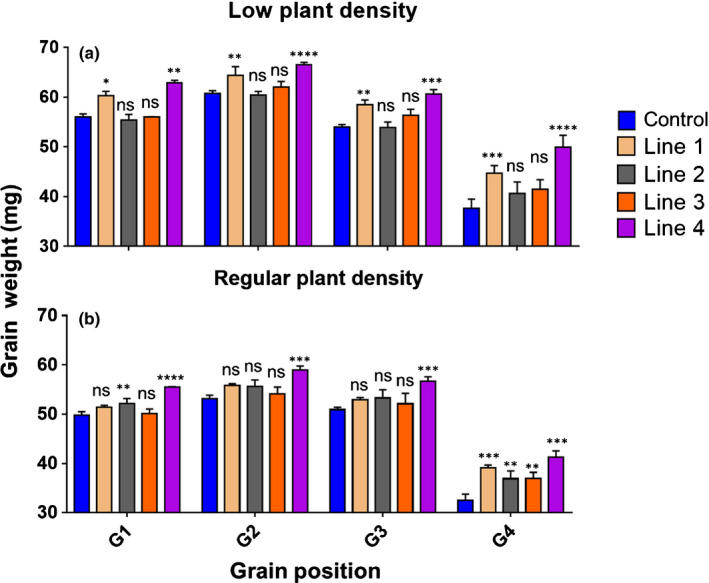
Individual grain weight. Grain weight at grain position 1 (G1), 2 (G2), 3 (G3) and 4 (G4) in the control and *TaExpA6* transgenic lines 1, 2, 3 and 4 at (a) low plant density and (b) regular plant density. The control line corresponds to spring wheat cv Fielder that has undergone the same tissue culture process as the transformed lines. Asterisks indicate significant differences evaluated by pairwise comparisons between each line and the Control (Fisher’s least significant difference test *post hoc*): *, *P* < 0.10; **, *P* < 0.05; ***, *P* < 0.01; ****, *P* < 0.001; ns, not significant. All data are shown as mean and SE.

In our growth environment, each central spikelet of one ear of wheat typically accommodates four grains, named G1–G4 based on the relative proximity to the rachis of the spike. The more distal grains are typically the smallest, with G1 and G2 typically the biggest. The examination of individual GW from our transgenic lines revealed significant increases in all four grain positions of line 4 in the LPD experiment (Fig. 4a). Interestingly, the biggest GW increases were seen in the more distal position (G4), which showed increases of up to 32.8% in line 4 compared with control, whereas in G1 the increase was 12.3%, with similar values in G2 (9.5%) and G3 (12.2%). Similar but nonsignificant increases in GW were seen in the other transgenic lines.

The RPD field experiment was performed using T_4_ seed planted at 300 m^−2^, which is generally used by farmers for spring wheat in southern Chile. In this separate generation and independent field experiment, similar increases in individual GW were evident in the transgenic lines compared with the control. Transgenic line 4 again showed the best performance in this experiment, outperforming the control in terms of GW by 11.6%, 10.9%, 11.3% and 26.6% in G1, G2, G3 and G4, respectively, with the biggest increases seen in G4 (Fig. 4b). The other transgenic lines showed increased GW compared with control in the distal grain positions G4 and at G1, G2 and G3 depending on the line and experiment (Fig. 4a,b).

**Fig. 5:**
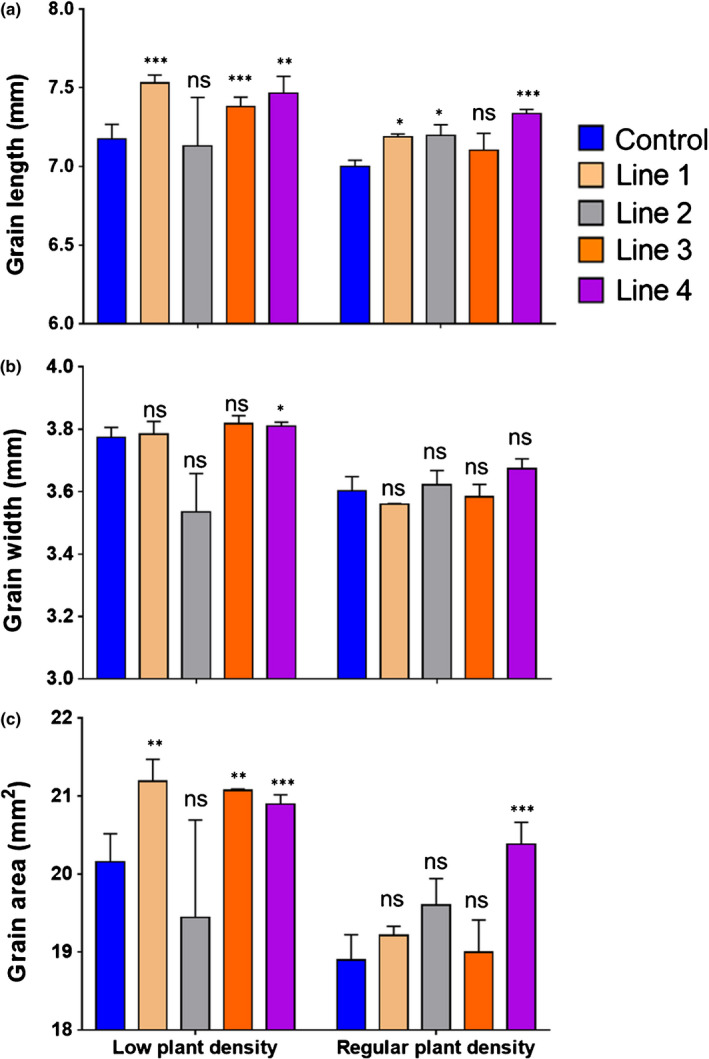
Grain length, width, and area at grain position 2 (G2) in wild‐type and transformed wheat lines. Grain dimensions were evaluated in control and four *TaExpA6* transgenic lines (lines 1–4) in field experiments at low plant density of 44 m^−2^ and regular agronomical plant density of 300 m^−2^. (a) Grain length, (b) grain width and (c) grain area. The control line corresponds to spring wheat cv Fielder that has undergone the same tissue culture process as the transformed lines. Asterisks indicate significant differences evaluated by pairwise comparisons between the control and each transgenic line (Fisher’s least significant difference test *post hoc*): *, *P* < 0.10; **, *P* < 0.05; ***, *P* < 0.01; ****, *P* < 0.001; ns, not significant. All data are shown as mean and SE.

**Fig. 6:**
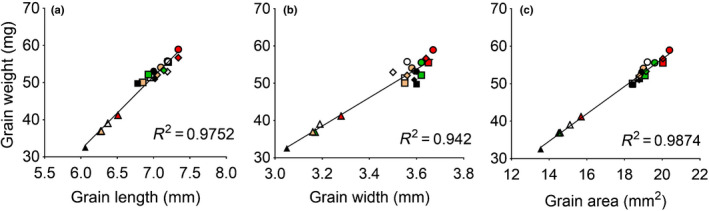
Association between individual grain weight and dimensions. Grain weight and (a) grain length, (b) grain width, and (c) grain area of grain positions 1 (G1: circle), 2 (G2: square), 3 (G3: rhombus) and 4 (G4: triangle) from central spikelets of the spike in the control (black) and transgenic lines 1 (white), 2 (green), 3 (orange) and 4 (red) recorded from the field experiment at plant density of 300 m^−2^.

Higher GW is likely associated with increased size. To examine this, we compared the length, width, and surface area of grains from transgenic and control lines from the RPD experiment. The results of grain dimensions of G2 (Fig. 5) showed that increased GW was most closely associated with increased grain length and area, with little increase in grain width; similar results were found in the other grain positions (Fig. S3). When the associations between GW and grain length, width, or area were plotted across genotypes and grain positions, higher consistency and better residual distributions were found for the relationship between GW and grain length (Fig. 6). This indicates that increased grain length is the largest contributor to higher grain area in transgenic lines.

### *TaExpA6* ectopic expression increases average grain weight and grain yield in transgenic wheat

**Fig. 7:**
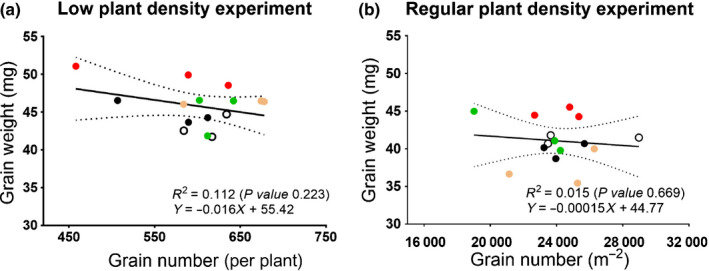
Trade‐off between grain weight and grain number. Relationship between grain weight and grain number of the control line (black circles) and transgenic lines 1 (open white circles), 2 (green circles), 3 (orange circles), and 4 (red circles) in the (a) low plant density and (b) regular agronomical plant density experiments. The regression line (continuous black line) and two confidence bands surrounding the best‐fit line that define the confidence interval (dotted lines) are shown.

The ectopic expression of *TaExpA6* increased average GW in the transgenic lines in both experiments, and this effect translated into increased total GY. Once again, the biggest increase in total yield was apparent for transgenic line 4, which showed the largest transcript and protein abundance of *TaExpA*6 during early grain development. GY per plant for line 4 was 9.5% higher than that of control, although this increase was not statistically significant (*P* > 0.10) in the LPD experiment (Table 1). However, GY was 11.3% higher than the control per square metre in the RPD field experiment (Table 2). In the RPD experiment, we monitored GY, GN and average GW for both stem categories (main stems and tillers). We observed no significant differences in total GN (*P* > 0.10) between control and transgenic lines, and the observed increases in overall yield (11.3%) are remarkably similar to those of individual GW in these experiments. These data reveal that there is no evident trade‐off between the major yield components (weight and number) in our experiments, as shown in Fig. 7. Notably, developmental phases and stages, such as dates of anthesis and physiological maturity, were similar among the transgenic lines and control in all experiments (Table S3). Line 4 was slightly taller (*P* < 0.01) than the control in the LPD and RPD experiments (Table S4). Line 1 also showed increased plant height, but only in the LPD experiment, whereas lines 2 and 3 did not show differences in plant height compared with control across experiments (Table S4).

**Table 1:** Grain yield (GY), grain number (GN), and average grain weight (GW) per plant in the control and transgenic lines recorded in the field experiment at low plant density of 44 m^−2^.

Wheat line	GY/plant (g)		GN/plant		GW (g)	
	Mean	SEM	Mean	SEM	Mean	SEM
Line 4	27.89 ns	2.29	561 ns	53.28	49.85 ****	0.73
Line 1	26.32 ns	1.05	612 ns	14.68	43.00 ns	0.89
Line 2	27.84 ns	1.23	619 ns	11.98	44.98 ns	1.56
Line 3	24.03 ns	0.69	501 ns	17.03	48.15 *	2.70
Control	25.47	1.02	569	32.00	44.82	0.88
						
ANOVA (*P*‐value)	0.359 ns		0.382 ns		0.016 **	
Line 4 and control (% difference)	9.5		−1.5		11.2	

All data are shown as mean and SEM. Control line corresponds to spring wheat cv Fielder that has undergone the same tissue culture process as the transformed lines. The phenotype data of each line was compared with control using the Fisher’s least significant difference test *post hoc*; asterisks indicate significant effects: *, *P* < 0.10; **, *P* < 0.05; ****, *P* < 0.001; ns, not significant. ANOVA *P*‐value and relative difference (%) for each trait between line 4 and the control is shown at the bottom of the table.

**Table 2:** Grain yield (GY), grain number (GN), and average grain weight (GW) per square metre of main stems, secondary tillers, and total in transformed lines and the control recorded in the field experiment at regular plant density of 300 m^−2^.

Wheat line	Main stems						Tillers						Total						
	GY (g m^−2^)		GN (m^−2^)		GW (mg)		GY (g m^−2^)		GN (m^−2^)		GW (mg)		GY (g m^−2^)		GN (m^−2^)		GW (mg)		
	Mean	SEM	Mean	SEM	Mean	SEM	Mean	SEM	Mean	SEM	Mean	SEM	Mean	SEM	Mean	SEM	Mean	SEM
Line 4	524 **	10.3	11 124 ns	347	47.1 ****	0.6	563 ns	30.3	13 148 ns	499	42.7 **	0.8	1086 *	39.2	24 272 ns	814	44.8 **	0.4
Line 1	533 **	42.5	12 533 *	972	42.5 ns	0.1	516 ns	45.2	12 836 ns	1127	40.2 ns	0.4	1049 ns	77.2	25 369 ns	1803	41.3 *	0.3
Line 2	464 ns	9.1	10 756 ns	374	43.3 ns	1.5	469 *	39.7	11 627 *	1359	40.7 ns	1.5	934 ns	39.3	22 382 ns	1684	41.9 ns	1.6
Line 3	459 ns	45.0	11 520 ns	950	39.8 ns	1.7	449 **	35.0	12 709 ns	634	35.2 **	1.1	907 ns	79.9	24 229 ns	1572	37.4 ns	1.4
Control	441	19.7	10 781	424	40.6	1.0	535	17.0	13 513	322	39.1	0.5	976	35.8	24 294	724	39.8	0.6
ANOVA (*P*‐value)	0.092*		0.270 ns		0.019**		0.039**		0.379 ns		0.010**		0.073*		0.455 ns		0.012**	
Line 4 and control (%)	18.9		3.2		16.0		5.1		−2.7		9.2		11.3		−0.1		12.3	

ANOVA *P*‐value and relative difference (%) for each trait between line 4 and the control is shown at the bottom of the table.

All data are shown as mean and SEM. Control line corresponds to spring wheat cv Fielder that has undergone the same tissue culture process as the transformed lines. The phenotype data of each line was compared with control using Fisher’s least significant difference test *post hoc*; asterisks indicate significant effects: *, *P* < 0.10; **, *P* < 0.05; ****, *P* < 0.001; ns, not significant.

## Discussion

The trade‐off between GW and GN has been reported in many studies (e.g. Foulkes *et al*., 2011; Molero *et al*., 2019 and references cited therein). From a physiological point of view, several studies have suggested that the negative correlation between GN and GW derives from the large proportion of ‘small grains’ at distal positions due to either wheat breeding or crop management and is independent of any competitive relationship among developing grains (Acreche & Slafer, 2006; Ferrante *et al*., 2015, 2017). This hypothesis is supported by the low correlation reported between final GW and starch‐synthesizing enzymes (Fahy *et al*., 2018). However, this does not explain why the successful attempts to increase GW have been accompanied by compensatory decreases in GN (e.g. Wiersma *et al*., 2001; Brinton *et al*., 2017; Wang *et al*., 2018). This negative relationship between weight and number was recently confirmed as a general phenomenon in analyses across a range of elite wheat genotypes (Quintero *et al*., 2018; Molero *et al*., 2019). A promising candidate gene was recently identified in wheat underlying a QTL that controls spikelet number per spike (Kuzay *et al*., 2019). Near‐isogenic lines carrying this gene increased GN, but GW concomitantly decreased by 19%, preventing an increase in yield. Few studies have made efforts to understand the molecular and genetic basis of the trade‐off between both main yield components in wheat. In tetraploid wheat, Golan et al. (2019) have suggested that GNI‐1 (Grain Number Increase 1) is involved in coordinating the trade‐off between GN and GW. In addition, trehalose6‐phosphate/SnRK1 has been suggested to influence GY by increasing the potential rate of filling and grain mass, but neither GN nor yield were reported by the authors (Zhang *et al*., 2017). Therefore, the key genes, time, and cellular location of this coordination remain largely undefined. This background suggests that it may be difficult to disrupt the complex regulatory pathways that control these crop traits. However, our results demonstrate that increasing the level of α‐expansin protein during early grain development leads to increased grain size in wheat as a result of increased grain length.

In our experiments, bigger grains resulted in increased total yield, as there is no associated compensatory decrease in GN. This contrasts with previous attempts to increase grain size using conventional wheat breeding, QTLs, or mutants (Wiersma *et al*., 2001; Brinton *et al*., 2017; Wang *et al*., 2018; Molero *et al*., 2019). Bae *et al*. (2014) reported increases in seed weight in Arabidopsis by the expression of a sweet potato expansin. In this case, expansin expression was driven by the broadly expressed cauliflower mosaic virus *35S* promoter and led to widespread morphological changes in the number and size of leaves, number of siliques, and so on in the plants. Since Arabidopsis is essentially a wild weedy plant, with no previous breeding for yield components, it is hard to draw any conclusions on agronomically relevant yield from those experiments. The use of a specific grain promoter in our work confined the expression of the *PinB::TaExpA6* transgene to the developing grain without detrimental pleiotropic impact on plant growth and development that might impair crop performance.

Our study reveals that it is possible to break the negative association between GW and GN using a targeted transgenic approach, at least in the high‐yielding environment of southern Chile. For many years, GN and GW were assumed to be independent of each other. However, it has been demonstrated that the developmental determination of these two key yield components shows a close temporal overlap in wheat, which occurs between booting and 10 DAA (Calderini *et al*., 1999; Ugarte *et al*., 2007; Brinton & Uauy, 2019). These findings were confirmed by a recent study of wheat cultivars across different environments in Australia (Parent *et al*., 2017). The overlap between the determination of GW and GN is similarly apparent in other grain crops, such as barley, triticale, sorghum, and sunflower (Lindström *et al*., 2006; Ugarte *et al*., 2007; Yang *et al*., 2009; Castillo *et al*., 2017). The temporal overlap between GN and GW determination suggests that they are developmentally linked, giving rise to the observed trade‐off between the two yield components before grain filling begins (Fig. 1).

Our targeted approach, using an early grain promoter to drive ectopic expression of an expansin gene in young developing grain, may have been successful due to the timing of the *PinB::TaExpA6* expression. The lack of expression of the *PinB::TaExpA6* at 5 DAA and the peak levels observed between 10 and 20 DAA in our study suggest that the fruitful increase of GW without a negative impact on GN was due to the expression of the expansin transgene occurring after the overlapping period of GW and GN determination, thereby avoiding the trade‐off between these yield components (see Fig. 1). However, only two of the four lines showed a significant positive impact on GY (lines 1 and 4), despite all four lines showing *TaExpA6* expression in the grain. The most likely explanation for this is that a threshold amount of additional expansin is needed in order to see a significant effect, as the lines with highest *TaExpA6* protein in developing grain showed the most significant increases in yield. It is, however, also possible that some of the differences between the lines may be the result of different transgene integration sites, which may impact on the expression of other genes in the region.

In our experiments, GY was increased in both LPD and RPD plantings, but the effect was greater at higher plant density. In the RPD experiment, we assessed the impact on grain in both the main stem and tillers and observed that the effects of the transgene were smaller in grains from tillers; that is, for line 4, GY of main stems increased almost 19% (*P* < 0.05) and tillers only 5% (*P* > 0.10). This may explain why GY was higher in the RPD experiment than when using the lower planting density, where more tillers are typically produced.

Our results are a very encouraging demonstration that the trade‐off between GW and GN can be broken and, as a consequence, GY can be increased. However, we recognize that more experiments across different environments and cultivars should be carried out to confirm the results presented here, which should be seen as a proof of concept of the positive effect of ectopic expansin expression in developing wheat seeds.

This work provides a simple approach for breaking barriers in wheat yield that may also prove important in a wide range of crops where the trade‐off between GN and grain size is widely observed.

## Author contributions

DFC conceived the project and coordinated the field trials, wheat transformation, experiments, and data analyses; FMC designed experiments and genetic constructs and analysed the experimental data; AA‐M evaluated expansin expression and molecular data analyses; GM and MPR collaborated in designing the LPD experiment and data analysis; MC, SB and MJM carried out the transformation of wheat; EJW supervised wheat transformation; AD performed the proteomic and data analysis for determining expansin abundance; LDG coordinated the experiments, evaluated transformants, and designed experiments, genetic constructs, and data analyses; SJM‐M conceived and coordinated the project, wheat transformation, experiments, and data analysis. DFC, FMC, LDG and SJM‐M wrote the manuscript with contributions from all authors.

## Supporting information


**Fig. S1** Schematic diagram of the binary plasmid pEW279‐Exp T‐DNA.
**Fig. S2** Screening of relative expression of transgene in 15 wheat transgenic lines at T_2_ generation.
**Fig. S3** Box and whiskers showing grain weight, grain length, grain width and grain area across grain positions (G1–G4) in control line and transgenic lines (1–4).
**Table S1** Selection criteria of four wheat transgenic lines to perform experiments at low and regular plant density.
**Table S2** Transgene copy number determined by NPTII amplification.
**Table S3** Phenology of transgenic wheat lines and control in both experiments.
**Table S4** Plant height of transgenic wheat lines and control in both experiments.
Please note: Wiley Blackwell are not responsible for the content or functionality of any Supporting Information supplied by the authors. Any queries (other than missing material) should be directed to the *New Phytologist* Central Office.
