# Supplementary material for: Overcoming the trade‐off between grain weight and number in wheat by the ectopic expression of expansin in developing seeds leads to increased yield potential
Source: New Phytol. 2020 Dec 4;230(2):629–40. doi: 10.1111/nph.17048 (PMC8048851; doi:10.1111/nph.17048)
Supplement: Supplementary file 1 — Fig. S1 Schematic diagram of the binary plasmid pEW279‐Exp T‐DNA. Fig. S2 Screening of relative expression of transgene in 15 wheat transgenic lines at T2 generation. Fig. S3 Box and whiskers showing grain weight, grain length, grain width and grain area across grain positions (G1–G4) in control line and transgenic lines (1–4). Table S1 Selection criteria of four wheat transgenic lines to perform experiments at low and regular plant density. Table S2 Transgene copy number determined by NPTII amplification. Table S3 Phenology of transgenic wheat lines and control in both experiments. Table S4 Plant height of transgenic wheat lines and control in both experiments. Please note: Wiley Blackwell are not responsible for the content or functionality of any Supporting Information supplied by the authors. Any queries (other than missing material) should be directed to the New Phytologist Central Office. [file NPH-230-629-s001.pdf]

***New Phytologist* Supporting Information**

Article title: Overcoming the trade-off between grain weight and number in wheat by the ectopic expression of expansin in developing seeds leads to increased yield potential
Authors: Daniel F. Calderini, Francisca M. Castillo, Anita Arenas-M, Gemma Molero, Matthew P. Reynolds, Melanie Craze, Sarah Bowden, Matthew J. Milner, Emma J. Wallington, Adam Dowle, Leonardo D. Gomez and Simon J. McQueen-Mason

Corresponding authors:
Daniel F. Calderini; danielcalderini@uach.cl
Simon J. McQueen-Mason; simon.mcqueenmason@york.ac.uk
Article acceptance date: 21 October 2020

The following Supporting Information is available for this article:

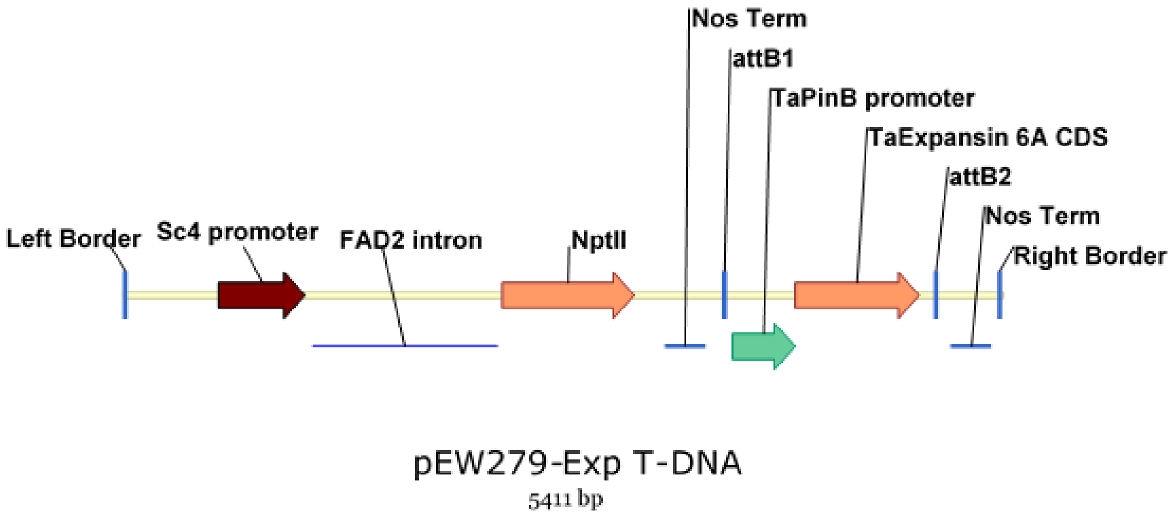

Fig. S1 Schematic diagram of the binary plasmid pEW279-Exp T-DNA.

Table S1. Selection criteria of four wheat transgenic lines to perform experiments at low and regular plant density

Wheat Lines	qPCR copy number (T0)	GW G1 (mg) (T2)	GW G2 (mg) (T2)	Yield (T2)	GW at harvest (mg) (T3)	average GW (mg) (T2) greenhouse	Copies_npt2_1 (T1)	Copies_npt2_2 (T2)	Screening of relative expression at 15DAA (T2)
52.16 (Line 4)	4+	62.9	66.5	240.0	57.6	62.5	4	4	11.36
52.4	4+	59.6	64.4	230.7	56.2	55.1	5	3	4.21
51.1 (Line 1)	4	60.3	64.4	227.9	49.9		2	2	6.05
51.18	4+	59.4	62.7	220.2	56.2		21	20	14.94
52.1	4+	55.7	62.3	212.6	50.2		4	3	4.66
52.11	4+	58.6	62.3	222.5	55.2		4	3	10.26
52.3 (Line 3)	4+	56.0	62.1	215.9	51.8		6	7	17.10
51.10	4	58.2	62.1	225.4	49.5		2	0	3.58
51.21	4+	57.2	62.0	217.8	49.1		4	7	5.77
52.12	4+	56.3	61.9	217.8	52.2		6	6	14.09
51.20	4+	56.7	61.7	213.4	50.8		5	7	10.86
52.14	4+	57.6	61.4	217.3	53.7		6	8	12.18
52.7	4+	56.5	60.7	214.4	48.3		4	4	15.58
51.14 (Line 2)	4+	55.3	60.4	210.2	48.3		4	8	11.16
51.con 1 (control)	0	55.8	60.2	208.8	51.3	52.5	0	0	0
52.2	4+	55.6	60.0		47.5		2	2	
52.8	4+	53.2	59.9		48.7		4	1	
52.15	4+	54.3	59.9		51.8		4	4	
51.9	4+	58.6	59.1		49.6		3	3	
51.6	4	54.6	59.1		48.2				
51.15	4+	53.2	58.9		49.1		15	16	
51.12	4+	57.8	58.7		48.4		0	6	
52.17	4+	52.9	58.7		50.8		8	4	
52.10	4+	53.1	58.2		47.4		0	0	
52.13	4+	53.4	57.1		48.9	50.0	13	nd	

All data are shown as mean of replicates. GW: Grain Weight, DAA: Days after anthesis, nd: not determined.

Table S2. Transgene copy number determined by NPTII amplification. nd: not determined

	NPTII gene copy number	
**Wheat line**	**Low density (T3)**	**Regular density (T4)**
51.1 (Line 1)	2	2
51.14 (Line 2)	6	12
52.3 (Line 3)	7	10
52.16 (Line 4)	4	4
51.10	2	
51.12	6	
51.15	16	
51.18	21	
51.2	6	
51.21	6	
51.9	3	
52.1	4	
52.10	4	
52.11	4	
52.12	4	
52.14	7	
52.15	4	
52.17	6	
52.2	2	
52.4	4	
52.7	4	
52.8	3	
52.13	13	
51.6	nd	
**51.con1 (control)**	**0**	**0**

Table S3. Phenology of transgenic wheat lines and control in both experiments. Dates of anthesis and physiological maturity (PM).

**Low density Experiment**		
wheat line	Anthesis	PM
Control	28-nov	12-ene
Line 1	28-nov	12-ene
Line 2	28-nov	12-ene
Line 3	28-nov	12-ene
Line 4	27-nov	11-ene
ANOVA P value	*0.832*	*0.772*

**Regular Density Experiment**		
wheat line	Anthesis	PM
Control	26-nov	17-ene
Line 1	26-nov	18-ene
Line 2	26-nov	17-ene
Line 3	26-nov	17-ene
Line 4	26-nov	17-ene
ANOVA P value	*0.978*	*0.941*

Table S4. Plant height of transgenic wheat lines and control in both experiments.

Low density Experiment

	**Plant height (m)**	
**Wheat Lines**	**Mean**	**SEM**
**Line 4**	0.90 ***	0.01
**Line 1**	0.86 **	0.02
**Line 2**	0.83 *ns*	0.01
**Line 3**	0.84 *ns*	0.02
**Control**	0.81	0.03
ANOVA (P value)	*0.029* **	

Regular density Experiment

	**Plant height (m)**	
**Wheat Lines**	**Mean**	**SEM**
**Line 4**	0.92 ***	0.00
**Line 1**	0.85 *ns*	0.03
**Line 2**	0.85 *ns*	0.01
**Line 3**	0.85 *ns*	0.01
**Control**	0.86	0.01
ANOVA (P value)	*0.017* **	

All data are shown as mean and standard error (SEM).
The phenotype data of each line was compared to control using the Fisher LSD test post-hoc; asterisks indicate significant effects at p<0.10 (*); p<0.05 (**); p<0.01 (***); p<0.001 (****); ns: not significant. ANOVA *P-value* is shown at the bottom of the table.

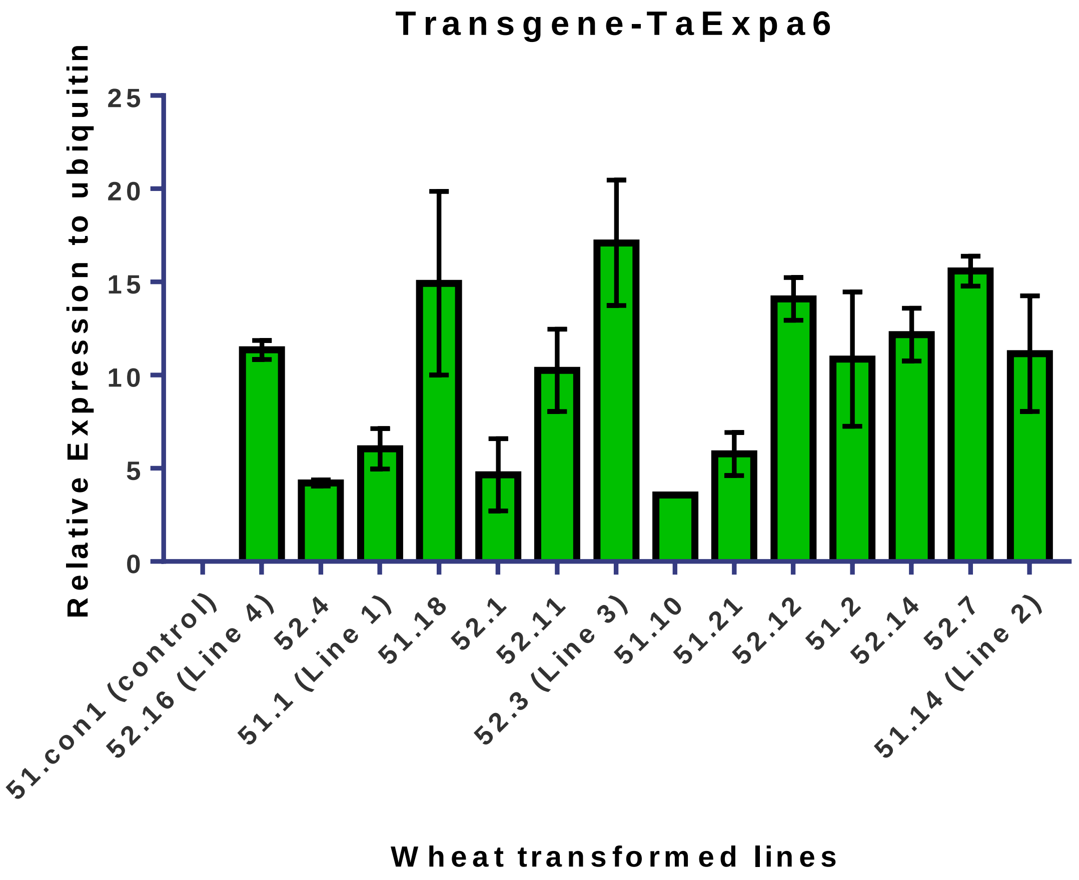

Fig. S2. Screening of relative expression of transgene in 15 wheat transgenic lines at T2 generation. All data are shown as mean and standard error.

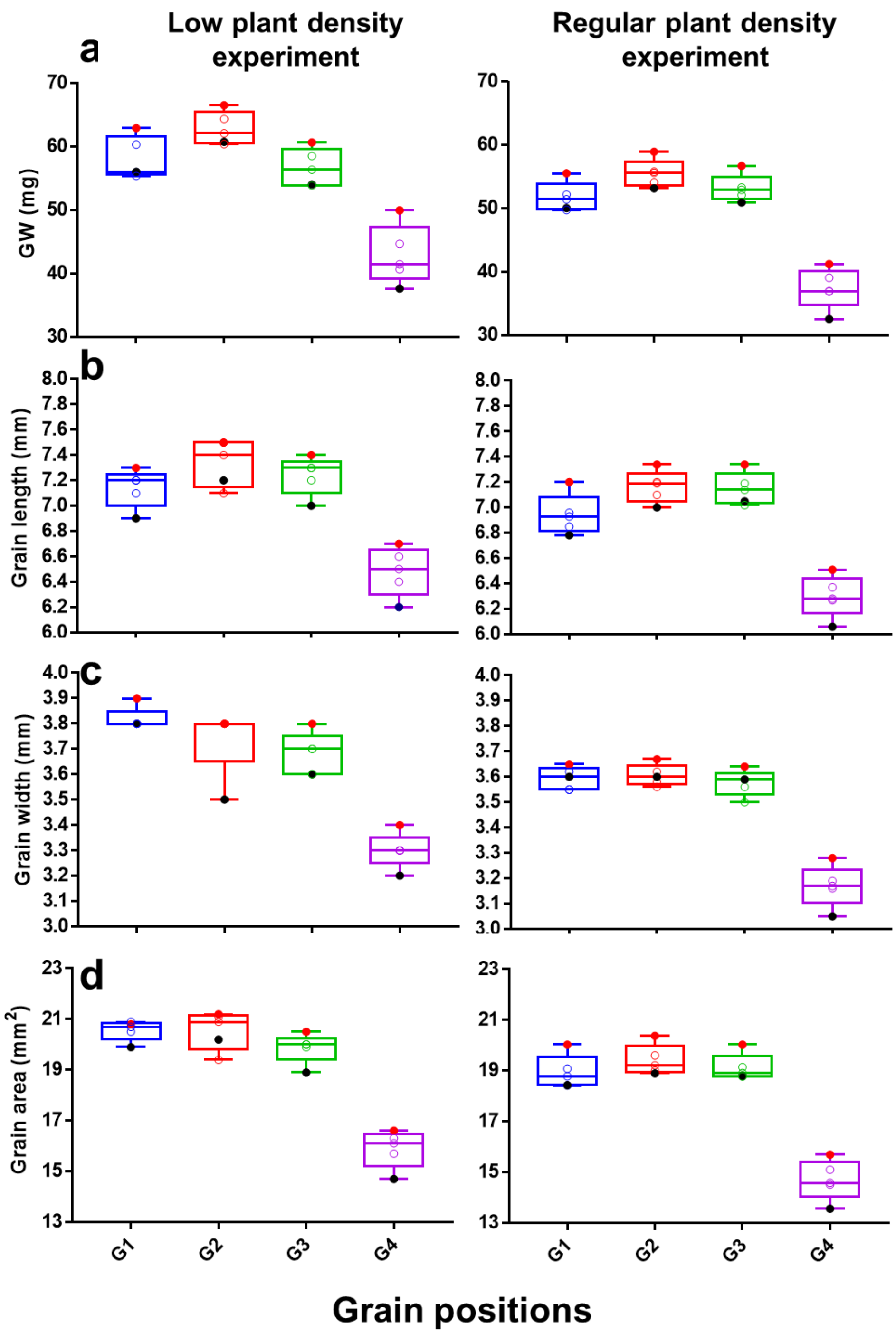

Fig. S3. Box and whiskers showing a) grain weight, b) grain length, c) grain width and d) grain area across grain positions (G1-G4) in control line and transgenic lines (1-4). In each graph the control line was identified with a black point and line 4 with a red point, open circles correspond to the wheat transgenic lines 1, 2 and 3. The horizontal lines in the box plots represent the minimum, 25th percentile, median, 75th percentile, and maximum values across the wheat transgenic lines and control line.
